# “Just Throw It Behind You and Just Keep Going”: Emotional Labor when Ethnic Minority Healthcare Staff Encounter Racism in Healthcare

**DOI:** 10.3389/fsoc.2021.741202

**Published:** 2022-01-12

**Authors:** Beth Maina Ahlberg, Sarah Hamed, Hannah Bradby, Cecilia Moberg, Suruchi Thapar-Björkert

**Affiliations:** ^1^ Department of Sociology, Uppsala University, Uppsala, Sweden; ^2^ Skaraborg Institute for Research and Development, Skövde, Sweden; ^3^ Department of Neurobiology, Care Sciences and Society, Karolinska Institute, Stockholm, Sweden; ^4^ Department of Government, Uppsala University, Uppsala, Sweden

**Keywords:** racism, healthcare, ethnic minority, staff, emotional labor

## Abstract

Encountering racism is burdensome and meeting it in a healthcare setting is no exception. This paper is part of a larger study that focused on understanding and addressing racism in healthcare in Sweden. In the paper, we draw on interviews with 12 ethnic minority healthcare staff who described how they managed emotional labor in their encounters with racism at their workplace. Data were analyzed using thematic analysis. The analysis revealed that experienced emotional labor arises from two main reasons. The first is the concern and fear that ethnic minority healthcare staff have of adverse consequences for their employment should they be seen engaged in discussing racism. The second concerns the ethical dilemmas when taking care of racist patients since healthcare staff are bound by a duty of providing equal care for all patients as expressed in healthcare institutional regulations. Strategies to manage emotional labor described by the staff include working harder to prove their competence and faking, blocking or hiding their emotions when they encounter racism. The emotional labor implied by these strategies could be intense or traumatizing as indicated by some staff members, and can therefore have negative effects on health. Given that discussions around racism are silenced, it is paramount to create space where racism can be safely discussed and to develop a safe healthcare environment for the benefit of staff and patients.

## Introduction

This article is about the ways ethnic minority healthcare staff manage emotional labor when they encounter racism at their workplace in Sweden. Research has shown that ethnic minority healthcare staff in different parts of the Global north experience both overt and covert racism from patients and others (Criddle et al., 2017; Moceri, 2014; Wingfield & Chavez, 2020). Healthcare staff describe stress and emotional depletion due to racism from patients (Cottingham et al., 2018; Eddo-Lodge, 2018) as well as in various medical education settings and in their workplaces (Arday, 2018). While research on racism in health and elderly care show that ethnic minority healthcare staff experience racism, other studies show that discussions around racism are silenced and are absent from organizational discussions (Behtoui et al., 2017; Bradby et al., 2019). Instead, complaints about racism by ethnic minority staff are trivialized and dismissed as there is lack of support from managers (Ngocha-Chaderopa & Boon, 2016). Ethnic majority nurses in the United States and New Zealand were, for example, punished for supporting ethnic minority nurses when the latter indicated they experienced racism in the workplace (Giddings, 2005). It appears that the experiences of racism by ethnic minority staff should just be tolerated (Moceri, 2014). This in turn has adverse effects which may include a loss of confidence in their medical abilities, feelings of isolation and exclusion from teamwork, and loss of job opportunities (Likupe & Archibong, 2013; Storm & Lowndes, 2021). Apart from studies focusing on ethnic minority staff’s experiences of racism in healthcare, many other studies have focused on the experiences of ethnic minority patients, showing various racial disparities in health and access to healthcare across national contexts and health indicators (e.g. Karlsen & Nazroo, 2002; Ben et al., 2017; Sim et al., 2021). Williams & Mohammed (2009) for example, note that for most of the 15 leading causes of death in United States, African Americans have higher death rates than whites. Anekwe (2020) too observed that researchers at Oxford University found that between 2014 and 2016, rates of death in pregnancy was eight in 100,000 for white women, 15 in 100,000 for Asian women and 40 in 100,000 for black women in the United Kingdom. Although racism in healthcare is complex and operates in various dimensions affecting both ethnic minority healthcare users and healthcare staff, this paper focuses on experiences of healthcare staff.

In spite of racial disparities mentioned above, medical professional practice values solidarity, equality, and scientific rationality highly. The insistence that healthcare is a rational practice of solidarity with the patient at the center (Judge & Ceci, 2021) acts as a hindrance to discussing the occurrence of racism (Hamed et al., 2020), and staff who express experiences of racism tend to have their concerns dismissed. This trivializing of racism can be seen as part of what Bain (2018) refers to as the practice of ignorance that, in turn, silences experiences of racism. Milazzo (2017) adds that notions of white ignorance, invisibility, privilege and shame, as theorized in critical philosophy of race, are however limited in the way they minimize white people’s active interest in reproducing the racist status quo. These practices of ignorance are moreover intertwined with practices of oppression and exclusion, which can, among those experiencing racism, translate into anxiety, fear, silence, and denial (Bain, 2018).

In this paper, we draw on interviews with 12 ethnic minority healthcare staff in Sweden in order to discuss how they said they manage their emotions when they encounter racism in their work. Before presenting our findings, we start with a short overview on racialized emotional labor, followed by a short description of the Swedish context and our methodology.

### Intersections of “Race” With Emotional Labor: Racialized Emotional Labor

The study of emotions has become central in sociology over the past decades (Bericat, 2016). According to Wharton (2009) sociological literature on emotional labor can be roughly divided into studies that use emotional labor to understand the organization, structure, and social relations of service jobs. Furthermore, emotional labor has also been used to understand the efforts of individuals to express and regulate emotions but also the consequences of those efforts. This paper focuses on how employees manage their emotions at the workplace. But while a great deal of research has focused on how emotional labor is gendered, it is, as argued by Humphrey (2021) also “raced”, although racialized emotional labor is an unseen burden among public-sector employees and this suggests a need to examine the intersection of race and emotional labor. It is from this perspective we use racialized emotional labor in this paper to reflect on how ethnic minority healthcare staff describe how they manage their emotions when they encounter racism at work.

Emotional labor according to Wharton (1999) is the process by which workers manage their feelings and emotions in relation to the organizational rules and guidelines. As conceptualized by (Hochschild, 2012), this emotional labor:

Requires one to induce or suppress feelings in order to sustain the outward countenance that produces the proper state of mind in others This kind of labor calls for a coordination of mind and feeling, and it sometimes draws on a source of self deep and integral to our individuality. (*p*:7)

It is also clear that emotional labor is understood differently by different analysts. Brook (2009) for example, has analyzed extensively how Sharon Bolton has criticized Hochschild’s notion that workers undergoing a “transmutation of feelings” renders them “crippled actors” in the grip of management control. Others have emphasized the need to understand emotional labor in different contexts. Erickson and Grove (2008) for example have examined literature on emotional labor in part to demonstrate how research has contributed in understanding emotional labor processes especially in increasingly changing economic contexts. Mirchandani (2003) also notes the need to understand emotion work entailed in contexts of workers’ heterogeneous social and economic environments, a point also emphasized by Chong (2009). While these are important ways of understanding emotional labor, this paper focuses, as already indicated, on racialized emotional labor where racial inequalities and racism are central.

In their study, Evans and Moore (2015) explored experiences of people of color in elite law schools and the commercial aviation industry in the United States, in part to understand the connection between white institutional spaces, emotional labor and resistance. They found that people of color in these institutions experience an unequal distribution of emotional labor as they negotiate both everyday racial micro-aggressions, but also dominant ideologies that deny the relevance of ‘race’ and racism. Consequently, professionals of color actively look for ways to promote counter narratives, in part to protect themselves from stigmatization and minimize the risk of severe consequences. In similar ways, black men in university settings develop strategies to dissociate themselves from racism that tends “to ignore, trivialize, and reinterpret everyday racism” (Wilkins, 2012: 58). Behtoui et al. (2017) observed in their study of employees in elder care in Sweden that employees from Africa, Asia and Latin America stayed silent rather than exiting the workplace, even when dissatisfied. Understanding emotional labor in the context of this silence around and silencing of racism as we elaborate below, is the aim of this paper.

Silencing of experiences of racism has been conceptualized as part of an epistemology of ignorance, although Sullivan and Tuana (2007) ask how such diametrically opposed concepts can go together, given that epistemology is about how one knows and ignorance is a condition of not knowing, such that epistemology should have nothing to do with ignorance. In answer to their own question, they argue that the epistemology of ignorance is a complex phenomenon of ignorance aiming to identify different forms of ignorance, how they are produced and sustained as well as the role they play in knowledge practices. As articulated by Mueller (2017, 2020), epistemology of ignorance is a system of ignoring and misinterpreting that reinforces white domination by white interests evading and distorting racial reality and racial injustice. For instance (Martin, 2021), illuminates how critical race theorists and the law are applying the epistemology of ignorance to issues of race, racism and white privilege to explore how forms of ignorance operate to enable racial oppression and domination in the United States. Thus, in the case of racism, the epistemology of racism is an epistemic practice of active knowing designed to produce not knowing about white privilege and structural white supremacy; a denial or active ignorance of a history of domination and of injustices committed in the interests of white people. This ‘willful ignorance’ is maintained by the ignorance of perspectives that challenge the prevalence of ignorance. In the context of whiteness, willful ignorance, is the cultivation of a stance in which the white self is allowed to consider itself as morally pure and untainted (Proctor & Schiebinger, 2008; Trepagnier, 2010; Martín, 2021), a position embodied in European exceptionalism. Eddo-Lodge (2018) describes this ignorance as an emotional disconnect where a vast majority of white people refuse to accept the existence of structural racism and its symptoms. According to Charles W. Mills (2015), silencing practices and experiences of racism relate to what he referred to as the:

Epistemology of ignorance that is “meant to denote an ignorance among whites – an absence of belief, a false belief, a set of false beliefs, a pervasively deforming outlook – that [is] not contingent but causally linked to their whiteness (*p*:217).

As articulated by Kendi (2019), the epistemology of ignorance is the failure to identify racist inequalities and disparities created through history. Scheurich and Young (1997) use the term ‘epistemological racism’ to categorize four levels of racism including: individual (overt and covert), institutional, societal and civilizational racism; the last of which they argue is the deepest level arising from the modernist period when European colonial and territorial expansion was undertaken under the rationale of the supremacy of white civilization. In other words, white supremacy became interwoven into the fabric of modern western civilization from the outset. The production and reproduction of racism significantly relies on cognitive and epistemological processes that produce ignorance, that in turn promote various ways of ignoring both the histories and legacies of European imperialism, as well as the testimonies and scholarship of those who experience racism in their everyday lives (Mills, 1999). But as argued by Sullivan and Tuana (2007), ignorance is not just a tool of oppression by the powerful, since it can be used for the survival of the victimized and oppressed as was the case with black slaves’ feigned ignorance of their masters’ lives. It can also take the form of the oppressed combating their oppression by unlearning the oppressor’s knowledge, whether it is passively absorbed or actively forced on them. This may explain some of the observations in our study where the staff manage their emotions on encountering racism, by just wanting to show how professional they are.

This silencing has effects on those experiencing racism, both material and emotional (Cottingham et al., 2018; Eddo-Lodge, 2018). The fear and anxiety which became apparent in our research and are described below, constitute an ‘emotional response tied to existing lives, their topographies, histories and daily insecurities’ (Pain, 2009: 478), and frames how social realities are understood, and, perhaps more significantly, how they are managed. It is in this context, we argue that anxieties expressed by ethnic minority healthcare staff, and more importantly how they coped with those anxieties, can be understood, as emotional labor. How racism plays out in healthcare settings may also have been further complicated by fear amplified through the current discourses around the ‘war on terror’ (Pain, 2009) which has augmented a politics of fear with direct implications for racialized minorities who are equated with being a threat to European society (Younis & Jadhav, 2019). This article examines the narratives of ethnic minority healthcare staff in relation to their emotional labor in response to encounters with silenced racism embedded in an epistemology of ignorance that exists in Swedish institutions, including healthcare (Alinia, 2020).

### The Silencing of Racism in the Swedish Context

We present in this section, an overview of the Swedish context of our research to demonstrate how the particular setting contributes to the silencing of racism. An open discussion about racism in Sweden is difficult in most institutions, including healthcare (Alinia, 2020). This is partly due to Sweden’s self-image as an equal, antiracist, human rights defender and a haven for refugees (Bäärnhielm et al., 2005). This self-image has its roots in the 17th century idea of the “hyperborea”, a Nordic version of eurocentrism, which enabled Sweden to have a double moral advantage in relation to colonization. On the one hand, Swedes could claim superiority vis a vis colonized peoples and on the other, as impartial explorers “in service of science and culture” (Schough, 2008, 36–38, 52), they could distance themselves from other colonizers (Björkert & Farahani, 2019; McEachrane, 2018). This moral high ground has been reinforced through the social and political movements of the 1960s and 1970s, when Sweden emerged on the international scene as a model of solidarity and equality, where decolonizing and anti-apartheid movements were widely supported, in the context of a strong welfare state identity (Pred, 2001). Furthermore, Sweden has been among the most generous European countries towards refugees (Hübinette & Lundström, 2014) at least prior to 2016, at which point a more restrictive refugee policy was put in place (Migrationsverket, 2016). On the other hand, Sweden’s role in the production of racial biology during the 19th century for example, when Carl Von Linnaeus divided humans into four distinct races and Anders Retzius developed methods of measuring skull of “different races, is not widely discussed in Sweden (McEachrane, 2018). Nonetheless these ideas helped to cement the idea of racial biological differences around the western world. The term “race” itself, was however removed from the Swedish law when, in 1973, the Swedish government argued to the United Nations that it was unnecessary to have laws against racism as the majority of Swedish people were regarded as anti-racist (Hübinette & Lundström, 2014). Later, in 2014, the Integration Minister argued that the removal of “race” from the legal statutes would help Sweden steer away from xenophobia (Mulinari & Neergaard, 2017), which effectively permitted institutional racism to persist, unchallenged.

There is thus no statutory data concerning ethnicity or ‘race’ in Sweden (Bradby et al., 2019). Using critical race theory and white ignorance studies, Alinia (2020) has examined the Swedish government’s policy document to reduce and prevent gender violence against women. Her analysis highlights how, by focusing on gender violence alone, the knowledge produced ignores and excludes racial and ethnic power structures or the ways they intersect, thus further producing, maintaining and normalizing racial otherness and specific forms of social exclusion. Yet racial discourses, according to Alinia (2020), are concealed in the way the concepts of culture and ethnicity are used for example, to excuse gender-based violence among migrant communities as driven by minority cultural beliefs and behaviors. In a similar way, Schömer (2016) has revealed the paradoxes in discriminatory structures in the Labor Court in Sweden by comparing the decisions made on cases of discrimination at the work place. Schömer found that the cases of racism against African workers, were dismissed while cases of gender discrimination were taken into consideration. African workers who reported being discriminated against experienced negative consequences in the workplace including being assigned a lower position and a salary reduction. Discussions around racism in Sweden are thus difficult and white ignorance has been able to develop and flourish, as processes of racialization are presumed irrelevant.

In healthcare, for the most part, despite being a discussion on discrimination, racism is absent as a category of discrimination. This further silences racism and renders it illegitimate and unspeakable. Research suggests that ethnic minority healthcare staff find it difficult to discuss their experiences of racist discrimination within the workplace (Salmonsson, 2014) and this affects how they respond to racism. A study of ethnic minority medical students shows that students suppress their everyday experiences of racism due to a lack of suitable space for discussing them (Kristoffersson et al., 2021). It is this climate of silence and the need to address this silence so as to undo the harms of racism that our research aimed to address. Sweden’s universal public healthcare system was subject to a policy change in 2010 (The Primary Healthcare Choice), to open it up to private provision and to allow patients to choose their own doctor and clinic. This “patient choice” seems to have led to discrimination against medical healthcare professionals with foreign-sounding names, as was recently exposed in the Swedish daily newspaper Dagens Nyheter (DN) (Adrian Sadikovic, 2021). Journalists, posing as patients who had recently moved to a new neighborhood, called 120 healthcare clinics and requested that their new doctor be an ethnic Swede. A total of 51 clinics responded positively to the request, 40 refused and only a handful explicitly said the request was unacceptable. Choosing or preferring an ethnic majority Swedish healthcare provider by Swedish patients was evident in our study.

### Research Methods

#### Participants and Recruitment

This article is based on the accounts of 12 of the 58 healthcare staff (N = 35 interviews) of diverse professional and ethnic backgrounds that we interviewed using a semi-structured interview guide between 2017 and 2020 and before the advent of the Covid-19 pandemic. These 12 were the staff who described having managed emotions when they encountered racism at work. Healthcare staff were recruited from various urban and rural areas in Sweden, both primary and tertiary care units, to investigate their views on racism as well as understand their experiences of racism. Table 1 below includes some of the characteristics of the 58 interviewed healthcare staff. The locations from which the healthcare staff were recruited will not be disclosed, to avoid the risk of identification, especially for those recruited from care units in small towns. Individual interviews (N = 30), focus group discussion (N = 3) and paired interviews (N = 2) were conducted. Most of the interviews were individually conducted in line with healthcare staff preferences, due to the sensitivity of the topics. Two interviews were conducted with a pair of people who already knew each other. In three cases healthcare staff were recruited from the same workplace and were willing to be interviewed together in a focus group discussion.

**TABLE 1:** Occupation, ethnicity and gender of the 58 healthcare staff that were interviewed (N = 58).

	N (%)
Occupation	—
Nurse	20 (34%)
Physician	11 (19%)
Dental professional	8 (14%)
Midwife	4 (7%)
Psychologist	3 (5%)
Other professions (Pharmacist, social worker, nurse aid, lab analyst and public health staff)	12 (21%)
Ethnic group	—
Ethnic minority	22 (38%)
Ethnic majority	36 (62%)
Gender	—
Female	46 (79%)
Male	12 (21%)

We had some challenges in recruiting research participants for this project. Consequently, we used various avenues including previous contacts within healthcare that project members had from previous research activities or from previous experience working as healthcare staff to facilitate recruitment of research participants. It was especially challenging to recruit ethnic minority healthcare staff. As seen in supplementary table 1, most of those interviewed healthcare staff belonged to the ethnic majority group (N = 36/58). The reason for this difficulty was not our inability to identify ethnic minority healthcare staff, but rather their unwillingness to share their experiences and views of racism with us. The major reason expressed for this unwillingness was the anxiety associated with discussing racism in Sweden. Moreover, ethnic minority healthcare staff were afraid that discussing racism with us would jeopardize their employment, even when we assured them of anonymity. Even when they agreed to be interviewed, some did not want to be interviewed at their workplace as they did not want to be seen with us, in case someone from their workplace would ask who we were.

Most of the interviews were in Swedish but some were in English and Arabic; two main languages spoken by the first and second authors who conducted most of the interviews and who are also of minority ethnic background. All the 12 interviews included in this analysis were conducted by the first and second author. From these 12 interviews all but two were audiotaped. The 12 healthcare staff interviewed included physicians (N = 4), nurse (N = 1), nursing aide (N = 1), midwives (N = 4) dentist (N = 1), dental hygienist (N = 1). All the 58 interviews were transcribed, stored and coded in AtlasTi8, a data analysis software.

### Data Analysis Process

In this analysis process we used both deductive and inductive (Fereday & Muir-Cochrane, 2006) for the 58 qualitative interview data. A relatively simple coding scheme for the whole data set was derived, tested and modified by all the authors in collaboration, with all 58 interviews coded, using AtlasTi8 software for data analysis. For this analysis a further round of inductive coding was undertaken, with additional codes identified among ethnic minority staff who experienced racism in healthcare, then followed by further thematic analysis (Sandelowski, 2002), to explore the reasoning and experiences that staff described, during which emotional labor was identified as a feature across 12 of the interviews. Although all ethnic minority healthcare staff interviewed had experienced explicit and/or implicit racism from either healthcare users or staff or both, emotional labor was identified by 12 of the 22 ethnic minority healthcare staff interviewed. This paper thus examines the emotional labor that ethnic minority healthcare staff reflected on undertaking when they encounter racism in the course of their work.

### Ethical Considerations

Ethical permission for the broader study was obtained from Uppsala Ethical Review Board (Dnr 2018/201). All the standard research routines were followed, to ensure informed participation. The study participants received verbal and written information about the study, their anonymity and confidentiality. Both verbal and written consent were obtained from participants, while prior to audio-recording permission was also obtained from the participants. All data are stored in accordance with the regulations of Uppsala University in password-protected files.

### Findings

From the data analysis, two themes regarding why ethnic minority healthcare staff hide or constrain their emotions when they encounter racism were developed. The first concerns anxiety over consequences to their work position while the second concerns the ethical dilemmas encountered when caring for racist patients because, irrespective of the violations or abuse, healthcare staff still have the duty to provide care for all those in need, while the ideal of patient centered care implies attending to patients’ own priorities. The ethical duty toward the patient was, moreover, strongly imparted during the education and training of staff. Ethnic minority healthcare staff indicated they had to work extra hard to prove that they were indeed competent. Except for a few cases, racist acts mostly consisted of micro-aggression and racial slurs, as described below.

### Emotional Labor and Anxieties Over Work Position

This section describes the different ways ethnic minority healthcare staff interacted with ethnic majority Swedish colleagues and the anxieties this provoked with regard to experiences of racism. Any discussion of racism at work was said to invoke anxiety over being reported to the boss, which was also said to be highly stressful. Immense pressure to appear as a ‘normal worker’ which meant avoiding to talk about racism, was reported. A nurse-midwife explained that although she was not afraid of us interviewing her at her workplace, she nonetheless had to be careful as there were colleagues who might report her to the boss for discussing racism. She explained how certain colleagues would check whether she has made a mistake simply because she is an immigrant and black, and that she has learnt to resist this by showing she is good at what she does. The threat of being reported to the boss was according to her, stressful because of the need to be on guard all the time. The emotional labor that was implied in resisting the threat of being reported was explained as follows:

But the thing is, as an immigrant, what I learnt early on is that you have to fight for yourself. You have to, if everybody is doing what you’re doing, you have to prove even more that I am good at this. Someone may report … some people might feel every little thing that you do, that they feel like: ‘Okay I’m going to tell the boss that this thing is happening.’ And then you feel like you have to be extra careful everywhere. You have to be extra careful. You know, just to not try to take place, too much. Just try to do what you are paid to do, try to listen to others and sometimes, may be do not express yourself as much.

Another ethnic minority midwife similarly indicated how she blocks herself when she experiences racism from other midwives. The reason for blocking herself or her experiences of racism is the lack of space for discussing racism and the anxiety over the repercussions from colleagues and management. Blocking one’s emotion when encountering racism is a clear case of emotional labor that can be especially stressful as racism may be an everyday experience. She said:

Anyway, for me as a person, I block myself. I stop listening to you. When you talk it goes in, but I, I just leave it. I don’t take in anymore, you know.

General practitioners (GPs) and surgeons described the way colleagues, particularly those junior to them in the organizational hierarchy, may attempt to take over their duties or report them to the boss for any small mistake. In one case, a GP noted during an interview that took place at a café, that being watched and reported to the chief, especially by junior colleagues – nurses and nurse aides – is regular and is very stressful for him. This participant reported an incident where he mistakenly double-booked a patient. Instead of talking to him, the nurse just sent the patient straight to the clinical unit manager. He said at the end ‘being watched is extremely stressful’ especially because he also has to hide his emotions from his white colleagues.

In another case, a GP who was in charge of a clinic that includes the emergency and ambulance care sections, explained at great length how the nurses tried to take over his position in the following way:

When the ambulance came, the nurses started talking and explaining in a way that seemed to indicate that they wanted to take over my role. They did not want me to explain what needs to be done and this was not comfortable. This happened twice and it seemed like they did not want to have me there. It felt like they did not want me here. I was therefore forced to talk to the chief about what had happened. He asked me whether I wished to lodge a formal complaint, but I said no and added that he should talk to the nurses and hear their side of the story. The chief doctor then talked to the nurses, and in turn also informed the one above him. They then talked to the nurses and then we all talked together. Still one of the nurses said she thought I could not speak Swedish although to get to my position one has to have proper Swedish and it is indeed the main criteria to get such a job. The other nurse said she thought I had no experience.

On the question of whether he was satisfied with the intervention he said:

Yes, also because the chief had asked me whether I wanted to tell the nurses what I felt, which I did.

Although the case was taken seriously by the manager (referred to as the ‘chief doctor’), it was handled as a one-off incident and did not generate any guidelines or protocols against racism in the workplace. Failing to institute clear guidelines on racism may further silence racism thereby making it even more invisible.

Another GP reflected on the lack of space to discuss racism encountered from both colleagues and patients and then noted how she behaves when she encounters racism:

You know, we face a lot of people like this every day. So, you should never take it personally. Just, throw it behind you and just keep going, because at the end of the day, if you are going to take everything personally, you are going to feel very bad about it.

She went on to explain how the lack of space for discussing, let alone reporting, racism can be traumatizing for ethnic minority healthcare staff:

I think, there does not exist that kind of space. And I think a lot of people of color … or healthcare staff are traumatized by these situations. And they just keep going, because yeah … I mean, you can ask probably every person of color, who work with patients. I mean, it would be absolutely surprising for me, if any of them going through a racist situation that does not really affect them. But some of them…. they came to the conclusion that: “Okay, if no one cares, I will as well not care.” And that makes the problem very normalized.

A midwife, who had presented herself during the interview as a strong personality, nonetheless elaborated the quandary and consequent emotional labor involved in her work. She described how she sometimes has to listen to her colleagues talking negatively about patients who are migrants from Africa and the Middle East. The midwife talked about how she is sometimes asked to interpret for patients from Somalia, as she is originally from Somalia herself. She described how what she hears in the room as an interpreter was upsetting because patients were not being treated properly:

I have interpreted for Somali patients because I speak Somali. And many times, as an interpreter, I am not allowed to say anything, or I am not allowed to have an opinion. I just have to interpret what is said in the room. But many times … it eats me up as a person. I have stopped interpreting because I see how these people are treated in healthcare.

In this quote the midwife talks about how it eats her up to listen to other healthcare staff talk negatively about Somali patients and how she witnesses the inadequate treatment they get. When asked if she ever discusses these issues in her workplace she responded:

No, you do not dare to talk about it in Sweden. Absolutely not. One must absolutely not mention the word racism or comment on something … you know, racist. You have to find other ways of talking about it.

The midwife stressed how she has to control her emotions by appearing professional for a whole day at work, and to appear strong and as though she is not affected by the racism she encounters or the racism that ethnic minority patients encounter. The emotional labor of maintaining her professional face as a competent midwife, despite witnessing racist practice was draining. As the issue of racism was silenced and not named as a category of discrimination, racialized healthcare staff are left to deal with racist experiences on their own, however painful and heavy.

### Ethical Dilemmas in Taking Care of Racist Patients

This section focuses on the dilemmas of taking care of patients who may be overtly or covertly racist towards ethnic minority staff who, due to a professional duty of care, learned during their training, and expressed in organizational rules, cannot refuse to care for such patients. A major concern for the patients was whether the healthcare staff they were meeting were qualified, and therefore knew what they were doing. Patients meeting black staff or staff who spoke Swedish with a foreign accent could express a wish to be cared for, by what they described as ‘proper’ doctor or nurse.

An ethnic minority midwife talked about the ethical dilemmas arising from the duty of caring for patients who are hostile. She described an incident where a patient refused to be taken care of by her, so the senior midwife in charge swapped her for another midwife, while telling her not to take it personally. The midwife then explained the ethical dilemma of meeting patients who do not want to be cared for by her as follows:

Yes, it has happened … I thought, as a midwife I have a duty … And I cannot say: ‘No, I do not want these patients.’ But I also felt when I came into the room, that these people will demand a lot of energy from me. So, I tried to prepare myself mentally, that the 8 hours I have in front of me will be tough, but I have to be professional. Because I noticed right away that I wasn’t wanted in that room, but I thought, I have to be professional.

An ethnic minority GP who was at the time of interview also a doctoral student elaborated on how the education of healthcare staff imparts ethics for medical work, stressing that the patient comes first. The GP doctoral student reflected on this in the following way:

I think there is a lot of ignorance among many people who work with healthcare about racism and how they deal with it. Unfortunately, that even includes people of color, because we have like we have been brainwashed somehow Because every time, we are reminded during all the years of education that the patient comes first. And you have to be understanding and supporting, which is a part of being a doctor. And if something happens, and the patients say anything, you should never take it personally. You could just imagine they have a bad day. But it feels like a way to normalize a certain behavior of patients or actually stop paying attention to the problem of racism that happens on a daily basis.

She then described a case where a patient did not want to be cared for by her, but also how her supervisor reacted. The patient said:

*“*I don’t want a n****r to take my blood!” It was an old person, and in that situation, like my supervisor said “Okay that was not nice! But I will take care of him.” So, I stepped aside. I was super sad and angry, because this is not…. if it happens outside my work, I will never shut up….

She went on to elaborate on how the supervisor continued to persuade her not to take the abuse seriously since it is so very common:

The patient came first. So, after they took blood and the patient left, the supervisor told me: ‘You know, we face a lot of people like this every day. So, you should never take it personally. Just, put it behind you and just keep going, because at the end of the day if you are going to take everything personally, you are going to feel very bad about it.’ But I was crying, because I, in a normal case, I would be very … like, I would be angry, and I would speak about it. And don’t forget as well, being a student, means that you have a very low … you are at the bottom of this hierarchy. So, we don’t have a lot of power within that. So, I have to accept it and swallow it, after my supervisor … which is not easy, and then afterwards, it’s just like people continue.… it’s very depressing actually, because when you go home, you are just totally damaged of this and angry*.*

According to this GP doctoral student, there is no space for discussing racism at the university or thereafter in medical practice, and this absence becomes a burden which she says can be traumatizing.

The GP presented earlier with the incident of nurses trying to take over his role, described his experiences after moving to a clinic where he said the majority of patients (80–90%) were ethnic majority Swedes. At this new job, he got a higher salary and was moreover near his home, which allowed him to spend more time taking care of his newborn baby. He nevertheless decided to move back to his previous clinic because as he said *‘*I felt like I was misplaced’ because the patient profile was so different from what he was used to. He described two particular encounters with patients after which he decided to move back to his old clinic, although as he stressed, they needed doctors in the clinic he was abandoning. One patient who had signs of pneumonia was suspicious and asked whether the GP was sure of what he was doing, implying he could not be a proper doctor. The GP had said to the patient:

You have pneumonia and we are going to take an x-ray … you are going to get antibiotics today. I am going to send you for an x-ray. When I get the results I will give you a call.

The GP explained the condition of the patient and why he made the x-ray decision in the following way:

If he hadn’t had blood in his sputum, I wouldn’t send him for x-ray. Because he had high inflammation profiles, like the things we take blood tests for, and when one listens to the lungs there are typical sounds that tells…. the history…. of the disease.

The patient, according to the doctor, had recently come back from Thailand and had high fever, and was coughing. The doctor explained to the patient that the x-ray would be done the same day and when the results were out, the doctor would immediately call the patient. It was at that moment when the patient asked the doctor:

Are you sure about what you are doing? If you are not sure, can any of your colleagues check on me?

The patient then stood up and went to request another doctor at the clinic’s reception, where he was directed to another doctor. The new doctor however told the patient he should do what the first doctor had said. Finally, the patient said he was going to visit a larger hospital instead. The GP noted that the patient seemed to want to consult any other doctor who was a majority ethnic Swede. The GP trivialized the patient’s refusal to accept his medical authority by saying the patient was not clear in the head.

During the same time, the GP further explained that besides micro-aggression from patients questioning his competence, a final stroke was the meeting during a single day of two patients with Swastika symbols tattooed on their backs. He explained that this was something he had previously seen only on TV, and after this he decided to leave the clinic even though they needed doctors, it was near his home and he had better terms of service, including a higher salary. In spite of these advantages, he felt this was not his place, which he described in the following way:

The funny thing … I have never seen I see on TV the swastika tattoos and In one day, there were two patients of mine with swastika tattoos. I have never seen this before. So, I gave my notice. ‘This is not my place.’ So I just left. But they need doctors, they need doctors.

In this case, the dilemma that seemed to weigh heavily on the doctor was his decision to leave the clinic despite knowing there was great need for doctors. This particular doctor seems to have feared for his own life in the face of patients tattooed with swastikas, given the symbol’s relation to historical as well as the contemporary racist violence which can be understood within the context of the increased politics of fear of migrants.

In another case, a GP who was also a surgeon described his encounter with a patient who did not want to be treated by what he termed as ‘svartskalle’ (literally ‘black head’ – a derogatory term for a racialized other) in the following way:

Once I had one of my colleagues, a nurse, follow me to my office. She said to me: ‘You have a patient waiting for you outside.’ He is on my list, I have to see him. So, I went to the waiting room and I called out: ‘Mr. X?’ Nobody. ‘Mr. X?’ Nobody? Alright, there are a lot of people waiting. So, I went back to my office, but the nurse came again: ‘Right, the patient is waiting for you.’ So, I went with her: ‘Mr. X?’ He told her: ‘I told you, I don’t want this svartskalle!’

The GP then asked the nurse to ask his colleague, an ethnic majority Swedish doctor, to take care of the patient in exchange for one of his patients. The patient had a bleeding hemorrhoid. The nurse informed the other doctor, what had happened, and the doctor then told the patient:

‘We have a good surgeon here, I want him to see you with me, if you are okay?’ He (the patient) said: ‘Okay.’ No, but he doesn’t know who this surgeon is. So, the doctor came to me and told me: ‘Alright, this is that patient, we need to see him together just a consultation, that’s it.’ Alright, okay, but I felt he can do that, my colleague. He can do, but he wants to treat this patient, and I cannot say no, because we both are doctors and we want to treat the sick patient, mentally and.…

The doctor who was rejected by the patient had to join the colleague in jointly treating the patient. The ethnic minority doctor was the more qualified for the condition the patient suffered from and, as he also argued, he could not refuse because, as a doctor he has a duty of care for the patient, no matter how offensive they are. Neither could he say ‘no’ to his colleague’s request to co-treat the patients, as this might have had other consequences.

In yet another case, a dentist explained how a patient blamed immigrants for being in Sweden and illegitimately consuming social welfare in the following way:

You take all our money and use it on those asylum-seeking children who have come here!

The dentist then told of another incident where another patient screamed at a nurse for being of foreign background, spat on her and told her she will never learn the language. According to the dentist, this abuse affected the nurse mentally and he added:

When it happened, I did not really know what to say or do. It happened so fast and she was really hurt and sad and we later sought psychological help, both of us, so as to talk about how this patient behavior has affected us.

The burden of abuse according to the dentist, is heavier because one cannot refuse to care even when insulted and discriminated against, because when one works in health or dental care, one works to help people, as the dentist noted:

I do not want to deny people care, especially when they come with acute need. I cannot say I do not want to treat you, which one should do when one has been treated differently and offended. But as a caregiver you cannot do it. You still want to treat because the person has pain, but it is difficult.

In another interview, a dental hygienist described how his patient always tries to dismiss him and spoke negatively about migrants and refugees as being lazy and welfare exploiters. He reports:

I do not usually pay much attention to what she does or says, but I know that the best way with such people is to just concentrate on the work. I’m doing my job, but next time she’s coming back to me.

Although the dental hygienist discussed how he tried to focus on his work, since one has to always take care of patients, he expressed how difficult it is for the same patient to come back to him and continue speaking negatively about refugees. Micro-aggression and racial slurs when directed at healthcare staff create, as discussed above, dilemmas since the staff have few choices except to adhere to the duty of care, which can be burdensome and demeaning, as the interview excerpts have indicated. A nurse-midwife similarly described how some patients directed their racial slurs. In one case an elderly patient said to her: ‘go back to your country … and stop using taxpayers’ money!’ Other times when in the corridor, she can hear patients saying they are looking for a nurse, but they do not talk to her and sometimes they do not want her to treat them. In response to such acts, she said she only works professionally, to show them she is a proper nurse. In this way she tries to defend herself against possible abuse within a context where she cannot refuse to take care of racist patients.

In another case, a nurse aide described an encounter she had with an ethnic majority Swedish elderly patient. The elderly woman said: ‘This is my first time to sit next to a n****r.’ The nurse aide then excused this by saying that the woman had of course only seen Africans on television, where what is mostly presented is hunger and poverty in Africa. She then stressed that the woman had not seen the different shades of Africa as a large and varied continent. Then this nurse aide asked the researcher whether she could be expected to be angry with this woman, but even before the researcher could answer, she said: ‘I cannot be angry with her, but I can be sad’.

## Discussion and Conclusion

We have described the concerns articulated by ethnic minority healthcare staff in relation to their encounters with racism at work. Fear and anxiety over the consequences of being identified as a colleague who discusses racism inhibited the recruitment of participants for our research, because the ethnic minority healthcare staff were anxious about jeopardizing their employment and risking good relations at work. The experiences of ethnic minority healthcare staff in their interaction with colleagues at the workplace and their encounter with racism while caring for ethnic majority Swedish patients, was described as complex. Many remained on guard, working hard to demonstrate their professional competence, but also because their duty is to care for the sick however, offensive or abusive they may be. Healthcare staff felt caught in the need to do no harm, with the patient’s right to care even when being abusive. The regularity with which GPs, dentists and surgeons were questioned as to whether they knew what they were doing reflects white ignorance (Alinia,2020; Sullivan and Tauna 2007; Trepagnier, 2010; Mueller, 2017; Eddo-Lodge, 2018;, 2020; Martin, 2021) which, in combination with Swedish exceptionalism has, over the years silenced racism as discussed earlier.

Patients’ racial preferences, micro-aggression and racial slurs were said to be stressful for healthcare staff, and the resulting stress for example, led in the case of the dentist, to the need for psychological care, a point also articulated by Kimani Paul-Emile et al. (2016) in the following way:

For many minority healthcare workers, expressions of patients’ racial preferences are painful and degrading indignities, which cumulatively contribute to moral distress and burnout (*p*: 710).

The complexity of reactions and the emotions for those individuals experiencing and witnessing racism at work can perhaps not be well understood, let alone changed, without understanding the Swedish context, where racism continues to be ignored and even erased from legal statutes. This has a number of implications. While ethnic minority healthcare staff may have little chance to report work-based racial discrimination from colleagues or patients, those referred to as “the chiefs” (or bosses) were also constrained in how they could deal with racist patients. Such organizational constraints have not been addressed by race and ethnicity scholarship in Sweden as elsewhere, which has largely neglected the role of organizations in the social construction of race (Mirchandani, 2003; Ray, 2019). Some staff excused racism on account of the advanced age of the racist patient and advised the abused staff member not to take the abuse seriously, especially because it is a common phenomenon. They thus normalized the racism and further silenced those experiencing the racism. It is around this complexity that understanding the role of the epistemology of ignorance or white ignorance we addressed earlier becomes critical for understanding the effects of patient racial slurs and micro-aggression and the anxieties this generates among ethnic minority healthcare staff. While racial slurs were common, our interviews also document occasional overt racism from patients, including the use of the n-word, spitting and reference to immigrants as illegitimate consumers of social welfare, sometimes referred to as ‘welfarism’ (Bradby et al., 2019).

Healthcare staff who expressed anxiety about jeopardizing their employment if seen to be engaged in discussing racism, also described various strategies including working harder to prove one’s professionalism and competence, blocking or hiding feelings, that can be conceptualized as ways of resisting racial degradation and thereby protecting oneself emotionally from the damaging consequences of racism. The strategies represent an important aspect of racial resilience or resistance, enabling ethnic minority groups to participate in racially oppressive institutions while maintaining and valuing their human dignity (Evans and Moore 2015; Eddo-Lodge 2018). In the process, the strategies pursued by those encountering racism paradoxically further silence the discussion of racism and awareness of its effects. In this context and complexity, it seems that stringent methods of changing epistemological forms of racism are needed (Bhavnani, 2001). One way would be to initiate vigorous dialogues among scholars and other stakeholders at policy and managerial levels, but also create earnest integration in the education system including in healthcare. (This is an area our broader project has initiated and published in the form of interventions to strengthen nursing education to recognize and deal with racism (Bradby et al., 2021). In two Universities in Sweden, we have constructed and implemented an educational package among nursing students, as a method of initiating a discussion on racism in healthcare.)

This article does not aim to repeat the long history of race, racialization, and racism. However, since we understand racism as a silent and silenced phenomenon in Swedish healthcare, we hope to find ways of un-doing that silence, which implies that understanding the role of history in constructing race, racialization and racism is crucial, not least as part of healthcare education programs. Only when we grasp the phenomenon of fear, silence and denial, as expressed in our study, can we engage policy makers and communities in a dialogue about how to change. If the current anti-racist movement asserting that “Black lives matter” is anything to go by, it is clear that active ignorance, often protecting white supremacy, is at large. Moreover, besides the micro-aggression and racial slurs that reflect the silencing and rendering of racism invisible, it is not clear how global fears (such as the war on terror) (Pain 2009) or what Altheide (2003) refers to as the politics of fear, affect healthcare. While there was not much articulation of this fear, the way patients asked health care providers to go back home, to stop using welfare and stop providing care to refugees who have come to the country, can be seen as part of a discourse of fear of migrants by ethnic majority Swedes. Moreover, the fear articulated by a GP facing patients’ Swastika tattoos and his flight from the clinic, located in an area described as having 80–90% ethnic majority Swedish patients, can be understood as an illustration of how ethnic minority staff are affected by the politics of fear. The GP’s abandonment of the clinic is similar to what is reported by Stafford, 2010) about a surgeon in Germany who refused to operate a male patient when he discovered Swastika symbol on him. How this politics of fear, which is contextual, material and relational, is experienced by ethnic minority healthcare staff and how it is linked to the welfarist claims against immigrants however, remains to be explored in detail. Another issue is the way that fear around discussing racism in professional settings may hinder research. Although it is not clear how the politics of fear affected the healthcare staff in this study, it is important to note that many ethnic minority healthcare staff in this study refused to be interviewed, but this constitutes a separate publication.

## Data Availability

The raw and anonymised data on which this analysis is based may be made available on request.
