# Supplementary material for: “Just Throw It Behind You and Just Keep Going”: Emotional Labor when Ethnic Minority Healthcare Staff Encounter Racism in Healthcare
Source: Front Sociol. 2022 Jan 12;6:741202. doi: 10.3389/fsoc.2021.741202 (PMC8789661; doi:10.3389/fsoc.2021.741202)
Supplement: Supplementary file 1 [file DataSheet1.PDF]

Table 1: Participant characteristics (N=58)

	**N (%)**
**Occupation**	
Nurse	20 (34%)
Physician	11 (19%)
Dental professional	8 (14%)
Midwife	4 (7%)
Psychologist	3 (5%)
Other professions (Pharmacist, social worker, nurse aid, lab analyst and public health staff)	12 (21%)
**Ethnic group**	
Ethnic minority	22 (38%)
Ethnic majority	36 (62%)
**Gender**	
Female	46 (79%)
Male	12 (21%)
